# Editorial Chit-Chat

**Published:** 1872-10

**Authors:** 


### EDITORIAL CHIT-CHAT.

OUR CONTRIBUTORS.—Several of them failed to furnish us with their manuscripts in season for this number.

ATTENTION SIR KNIGHT.—The King of Sweden has conferred the knighthood of the Wasa on Dr. L. A. Sayre, of New York.

DR. NELATON, the eminent French Surgeon, is reported to be failing in health, caused by an internal tumor which cannot be reached or cured.

STRONG FIRM.—Gill & Gillmore is the name of a firm on Water street, dealers in tobacco. It is supposed the firm is composed of man and wife.

NEW ORDER.—The Knights Templar of this city are working a new order, called the English Right of Knighthood. It is to be a select affair.

A RARE BINDING.—A copy of the "Constitution of the French Republic of 1794," bound in human skin, is offered for sale in Paris. Some doctor should be the purchaser.

---

BUSY.—Major Luther Caldwell is kept busy looking after his wooden pavement. He has just returned from Indianapolis, Ind. When at home, he is most interested in laying the Greeley pave.

---

ACADEMY OF SCIENCE.—Is it not time for another meeting of this interesting society? Will not Prof. Ford wake up the president and have him tell us something about the chemical experiments with which his mind is so engrossed?

---

THE ARTISTS.—Conkey, the Sculptor, has returned to Chicago. Waters, the landscape painter, has been making studies of Lake George scenery. Chandler, of Philadelphia, is sketching at Ralston.

---

TYNDALL.—Prof. Tyndall, the great English scientist, is expected in this country during this month, and will lecture in our principal cities for the next eight months. Will not the Y. M. C. A. give us an opportunity to hear him?

---

FRASIER HOUSE, near the depot, is the place to stop during the State Fair. You are convenient to street and railroad cars for the fair grounds, as well as having an opportunity for eating at the best public table in the city.

---

IVY POISON.—Having recently suffered from poison from ivy, we were kindly furnished, by sympathising friends, with eighty-six well authenticated means of cure. We tried none of them, and are happy to announce our complete recovery.

---

NO GO.—A foreign vegetarian, who came to this country expecting to reap a fortune from manufacturing unbolted flour, has given it up as impracticable, because, as he asserts, the Americans give so little time to their meals that they bolt everything they eat.

---

SHOOTING.—A "gyro" pigeon club is being organized in this city. The pigeons are made of steel, and can be arranged to fly in imitation of pigeons, woodcock or snipe. They are thrown from a trap and are shot upon the wing, affording excellent sport for crack shots. It is more humane sport than to shoot live pigeons, just for fun.

MR. SMILLIE, an artist of considerable note, and a landscape painter of great genius, is, and has been, a guest at Gen. Diven's residence for the past few weeks, making sketches in and about our valley.

---

PROF. FORD has had two severe attacks of vertigo, necessitating complete rest from his scientific labors for awhile. We hope that a few weeks absence from his study will restore him to his usual vigor and health.

---

MR. CHARLES PALMER, of Brooklyn, was recently in our city, looking for an eligible site for erecting a foundry. We believe he has concluded to locate the establishment in Brooklyn, having just purchased a portion of his building materials in this place.

---

HOT.—We see it stated in our foreign medical journals, that the heat was so intense during July and August, in the northwestern provinces of India, that it was impossible for a European to leave the house after nine o'clock in the morning, and the birds took refuge in the houses.

---

SCRANTON.—The smallpox has been raging during the entire winter and summer in Scranton, Pa., and it seems no effort has been made on the part of the city authorities to arrest the spread of the contagion. We hear that the disease still exists there, and that occasionally a death occurs from it.

---

NON-COMMITTAL.—We have a lady friend who, when asked who her presidential candidate is, insists that she is for Gr——, but stoutly refuses to add another letter, by which her meaning might be surmised. In this respect she differs widely from many politicians, who are only too glad to offer a letter of explanation whenever an opportunity presents.

---

PROFESSIONAL LADIES.—A young German lady recently presented herself before the faculty of a Medical College at Munich, for an examination for a license to practice dentistry. Being refused, she went to Erlemgen, where the government authorized the examination, on the ground that it was absurd to exclude a person desirous of submitting herself to authorized professional tests of ability, by reason of her sex.—The young lady triumphed, and "is likely," it is added, "to find immediate imitators."

FINE PRINTING.—The finest specimen of job printing we have ever seen has just been issued from the *Advertiser* job rooms, by Mr. Robt. M. Watts. It is a card, about six by twelve inches, artistically designed, beautifully printed in colors, and is intended as a specimen of what can be accomplished in the office referred to. We learn it is intended for exhibition at the State Fair—it is well worth a close inspection.

NEW CITIZENS.—Those popular conductors of the Lehigh Valley Railroad, Messrs. Wm. H. Nicholas, G. Parks, Wm. H. and Chas. H. Cummings, expect to change their "runs" so as to have their "off days" in this city, instead of at Easton. We will be glad to number them among our citizens, as the Lehigh Valley Co are noted for the efficient and intelligent gentlemen whom they employ to manage their pass enger trains.

CUNDURANGO.—The death of the mother-in-law of Vice President Colfax, has greatly depreciated the market value of cundurango. Drs. Bliss & Keene puffed their "specific" into notoriety by claiming that the lady referred to, had been cured of cancer by the use of cundurango. The telegraph has been indiscreet enough to notify all the newspapers in christendom, that Mrs. Mathews died from cancer, hence the depreciation of the "great cancer specific."

SHARP ROGUES.—The rogues of New York have adopted an ingenious trick by which several have escaped from prison. A tombs prisoner obtained some croton oil and sprinkled it upon his hands and face, causing pustules, which looked so very like small pox, that he was removed to Bellevue Hospital, from which he easily escaped. Four others tried the experiment, but the trick was discovered and the scare subsided.

DEATH OF AN OLD FRIEND.—We see an announcement that N. B. Greene, Esq , of Fisherville, N. H., was recently thrown from a carriage, receiving a fracture of the skull and other injuries, from which he died. His daughter Kate, who was riding with him at the time, had her ankle broken. Mr. Greene will be remembered by many in this city and at Petroleum Centre, Pa., at which latter place he was interested with us in building the Oil Exchange Hotel. He was a railroad contractor by occupation, an honest gentleman and a true friend—peace to his ashes.

NEW YORK.—Elmira people will be glad to learn that Mr. Uriah Bartholomew, of this city, has taken the Rutledge House, at 834 Broadway, New York, and that it will be head quarters for Elmirans visiting the city in future.—The Rutledge House is kept on the European plan, furnished throughout in elegant style, where comfortable apartments can be had by the day or week, at reasonable rates. Call on Bartholomew when you visit the city.

THE LECTURE COURSE.—Dr. Hart informs us that the course for this winter will be made up from lectures by Geo. McDonald, Brete Harte, Collier, and Stanley—the finder of Dr. Livingstone. The readings will be given by Vandenhoff and wife, and Mrs. Scott Siddons. The musical entertainment by the Mendelshons, and the remaining lecture by either Gough or Anna Dickinson. This will make a very excellent course, worthy of liberal support.

THE IRON PRINCE.—Dr. Edwin Eldridge informs us that he intends building an immense blast furnace at Wistar Mountain, immediately. He deems the enterprise advisable by reason of his having coal, ore, and lime in great abundance at this point. The Doctor has not only the means, but also the energy and skill to manage such an enterprise, and we doubt not it will be a great success in his hands. The furnace which he has already erected near this city will be lighted and placed in operation within a few days. The Doctor never engages in small transactions, all his schemes are vast and costly, from which he reaps a corresponding amount of profit, to spend in princely sums, for the benefit of the people among whom he resides.

PHOTOGRAPHIC INVENTION.—A contrivance for photographic "swinging round the circle" is announced in England, and by a singular coincidence, its inventor's name is Johnson. The device is a panoramic camera, which by ingenious mechanism sweeps the whole landscape and takes it on a plane surface embracing on one negative one third of the circle. The exactitude of its operation is as singular as the beauty of its results. The pantoscope begins at one end of the view desired and goes round the horizon as one sweeps the telescope, the plate moving with a corresponding motion through the arc, which might be made a complete circle if it were desirable.

CHEAP ADVICE.—We saw the following recipe for the cure of a "cold in the head," in a newspaper the other day: "Take one teaspoonful of pine tar and one of pulverized sulphur, and burn it in an iron spoon, while you inhale the vapor." We wonder whether that editor ever took this sulphurous dose? If he has, perhaps it is his desire to suffocate his delinquent subscribers, but we would not advise any one who in the least enjoys this life, to try the "cure."

PERSONAL.—Ex O. Ficio—Ben. H. Pratt—the pleasant and entertaining writer, to whom the BISTOURY readers are indebted for many instructive and chatty communications, is in the city visiting friends.

P. C. Van Gelder, Esq., editor of the Wellsboro *Agitator*, has sold his newspaper establishment, and talks of purchasing an interest in the *Daily Advertiser*, of this city. Come along, Van—lots of room for as clever gentlemen as you.

A. L. Williams, Esq., Canandaigua's "dead shot," is visiting Mr. Hamlin, and the two are after woodcock in the Chemung and Susquehanna Valleys. The cocks can't dive quick enough to avoid these shots.

Hon. Frank Hall, has returned from his extensive travels abroad, and has settled down in our midst, impatient in the thought that one country remains unexplored by him. He is now occupied with maps of the regions about the north pole, evidently intent upon a visitation to that frigid territory at no distant day. No better or more entertaining lecture will be given in the course this winter, than could be furnished by Mr. Hall. We hope the lecture committee can prevail upon him to furnish our citizens with an account of his recent travels.

THE SUN.—Astronomers are just now very much interested in examining the spots on the sun. They startle us by announcing that that luminous body, to which we are indebted for our light and warmth, is absolutely devouring himself—burning himself up! They also terrify us by demonstrating to us, with mathematical precision, that in the course of a given number of thousands of years, the sun will be entirely destroyed, and our beautiful earth left in darkness and cold. We thus early notify our readers of what is to happen, so that they can lay in a large supply of coal and oil, and have their furnaces and lamps in the best possible condition for such an emergency.

THE BRIDGE PIERS.—The piers for the Main street iron bridge are to be built of solid and substantial masonry upon a foundation of oaken piles driven into the bed of the river and then sawed off below the surface of the water. The contractors claim that the timber will never rot while under water, and that if we will take the trouble to examine it a thousand years hence, it will be found to be as sound as when placed in position. We are afraid we will be unable to attend the inspection at the time appointed, and therefore our readers may never hear of the result.

AERONAUTIC.—Mr. Samuel A. King, the aeronaut, has been in our city endeavoring to make arrangements for an ascension, in his balloon "Aurora," during State Fair week. He is engaged in making a series of experimental ascensions for the signal service, being accompanied by Mr. Shaffer, of the United States Signal Corps, who carries with him all the instruments necessary to make scientific observations in the aerial regions. Mr. King expects to render the balloon of real service to science, in these observations, and we are glad to know that his endeavors are being appreciated and encouraged by the Chief Signal Officer.

FAMOUS.—Elmira is becoming famous throughout the State for its enterprise. Through the energy and liberality of its citizens, we have secured the location of the Reformatory Prison in our city, an immense structure, upon which millions of dollars will be expended, adding greatly to the beauty and importance of the place. Our city has also been designated as one of the permanent places for holding the State Fair. Eldrige Park is extensively known as one of the finest resorts in the State. Our school buildings, pavements, street railway, handsome residences, fire department and iron bridges, are the wonder and delight of visitors. We are constantly astonishing our neighbors with our manner of doing things; everything we have is upon a magnificent scale. Witness our two splendid brass bands in their brilliant uniforms and gorgeous drum majors—are we not justly proud of them? It is well worth a day's journey to Elmira to see her institutions, among which are classed her bands, and which can hardly be excelled in the State. We will match Asa's cornet and Joe's *baton* against all comers.

COZY.—When making the necessary repairs of your dwelling this fall, do not neglect putting in a grate in the living or family room, in which to burn coal. It is not only cheerful and comfortable, but is really a very valuable and effective means of ventilating the room. We have been examining the different styles of grates and mantels kept in this city, and recommend those furnished by A. W. Ayres, on Water street.—Nothing can tend more to make home cheerful and comfortable than a low-down grate, good wife and happy children. Ayres supplies only the grate, the wife and babies must be furnished by yourself.

---

A MAD DOCTOR.—A celebrated physician of Paris, who is widely known among the profession as an accomplished accoucheur, was recently taken with symptoms of insanity, and was therefore confined in the Charenton Hospital. A few days ago he was permitted to go to his banker's to collect some money. At the moment the cashier presented his head at the little door of his desk, the doctor exclaimed—"Bravo! the infant presents by the head!" and, seizing the startled cashier by the hair, tried desperately to deliver him through the opening. The cries of the victim brought help, and it was with great difficulty that the doctor was induced to give up his case.

---

TAPE WORM MANIA.—As it has been estimated, by an intelligent statistician, that one-half of our citizens are being treated for the expulsion of tape worms, and as the process is said to be somewhat debilitating, perhaps some of the anxious ones, who have difficulty in securing the monster, would be glad to know that a Dr. Stephen Myers, of Logansport, Ind., has patented a *trap* for catching the tape worm alive. We find this description of the article in the Patent Office Reports: "Trap of gold, one inch long by quarter inch in diameter, a hollow tube in three pieces, having holes and other contrivances." The directions for using the trap are as follows: "After the patient has fasted two to six days, the trap, baited for the worm, is swallowed, the end of a cord attached being returned from the mouth. The worm, in reaching the bait through a hole, is to get his head caught by teeth, when trap and worm are withdrawn together." If the worm is not caught in twelve hours, the trap is hauled up and baited anew. That is "a good one." The doctor should take up his residence in this city, and open a large manufacturing establishment for his traps, where a certain fortune awaits him.

---

FRENCH DIET.—The Paris correspondent of the *Medical Times and Gazette*, of June 22d, states that the Parisians are now taking bullock's blood for debility and consumption.—He says it is curious to see patients of all classes and both sexes, flocking to the slaughter-houses every morning to drink the freshly-drawn blood of the slaughtered animals. The young ladies take it readily, and many say they prefer it to cod liver oil.

This being the case, it is not improbable that Gilson will be drawing fresh bullock's blood, "from the skin," before long. Such hobbies are contagious—it only requires *fashion* to make it popular. We would not be surprised to see a fashionable young lady quaff her mug of fresh blood, should the Parisian sensation reach our shores.

